# Altered Distribution of Peripheral Blood Memory B Cells in Humans Chronically Infected with *Trypanosoma cruzi*

**DOI:** 10.1371/journal.pone.0104951

**Published:** 2014-08-11

**Authors:** Esteban R. Fernández, Gabriela C. Olivera, Luz P. Quebrada Palacio, Mariela N. González, Yolanda Hernandez-Vasquez, Natalia María Sirena, María L. Morán, Oscar S. Ledesma Patiño, Miriam Postan

**Affiliations:** 1 Departamento de Investigación, Instituto Nacional de Parasitología “Dr. Mario Fatala Chabén”, Ciudad Autónoma de Buenos Aires, Argentina; 2 Consejo Nacional de Investigaciones Científicas y Técnicas, Buenos Aires, Argentina; 3 Centro de Enfermedad de Chagas y Patología Regional, Hospital Independencia, Santiago del Estero, Argentina; Federal University of São Paulo, Brazil

## Abstract

Numerous abnormalities of the peripheral blood T cell compartment have been reported in human chronic *Trypanosoma cruzi* infection and related to prolonged antigenic stimulation by persisting parasites. Herein, we measured circulating lymphocytes of various phenotypes based on the differential expression of CD19, CD4, CD27, CD10, IgD, IgM, IgG and CD138 in a total of 48 *T. cruzi*-infected individuals and 24 healthy controls. Infected individuals had decreased frequencies of CD19+CD27+ cells, which positively correlated with the frequencies of CD4+CD27+ cells. The contraction of CD19+CD27+ cells was comprised of IgG+IgD-, IgM+IgD- and isotype switched IgM-IgD- memory B cells, CD19+CD10+CD27+ B cell precursors and terminally differentiated CD19+CD27+CD138+ plasma cells. Conversely, infected individuals had increased proportions of CD19+IgG+CD27-IgD- memory and CD19+IgM+CD27-IgD+ transitional/naïve B cells. These observations prompted us to assess soluble CD27, a molecule generated by the cleavage of membrane-bound CD27 and used to monitor systemic immune activation. Elevated levels of serum soluble CD27 were observed in infected individuals with Chagas cardiomyopathy, indicating its potentiality as an immunological marker for disease progression in endemic areas. In conclusion, our results demonstrate that chronic *T. cruzi* infection alters the distribution of various peripheral blood B cell subsets, probably related to the CD4+ T cell deregulation process provoked by the parasite in humans.

## Introduction

About 7 to 8 million people are infected with *Trypanosoma cruzi*, the causative agent of Chagas disease, with a further 25 million people at risk in Latin America [1]. Treatment against the parasite with currently available drugs leads to parasitological cure most acute infection cases, but its effectiveness on chronic infections remains uncertain [2]. Chronic infection can progress to end-stage cardiomyopathy and/or digestive abnormalities in up to 30% of infected patients, contributing significantly to morbidity and mortality in endemic areas.

The mechanisms underlying the development of chronic lesions are not fully understood [3]. Studies of the immune response against *T. cruzi* in humans have described numerous T cell alterations in the peripheral blood (PB) of infected individuals [3]–[6]. Some of the T cell defects that arise in chronically infected patients could be reverted by treatment against the parasite, indicating that they are likely a reflection of chronic antigenic stimulation driven by persistent parasites [7]–[9].

The potential involvement of B cells in the pathogenesis of chronic Chagas disease has been overshadowed by the emphasis on T cell research. Because B cells and plasma cells are barely found in *T. cruzi*-induced tissue lesions [10], [11] and most circulating B cells lack parasite specificity [12], [13], it is assumed that B cells play a secondary role in the control of *T. cruzi* infection [14]. However, increased percentages of circulating activated CD5+, HLA-DR+, caspase-3+ and granzyme B+ B cells were described in human *T. cruzi* infections and suggested to be involved in the development of Chagas heart disease [15]–[18]. Furthermore, experimental studies have shown that *T. cruzi* infection of mice lacking mature B cells results in increased parasitism, decreased inflammation and deficient generation of memory T cells, indicating their crucial role in the control of parasite multiplication and modulation of T cell responses [19].

To advance our understanding of the impact of *T. cruzi* infection on human immune responses, we here measured the levels of PB B cells of various phenotypes in chronically infected individuals. The study allowed us to identify a selective reduction of CD19+CD27+ cells in infected individuals, which correlated with reduced proportions of circulating CD4+CD27+ T cells. The contraction of CD19+CD27+ cells comprised IgG+, IgM+ and isotype switched memory B cells, B cell precursors and terminally differentiated plasma cells. These observations raise a question as to whether declining in circulating CD27+ cells affects the generation of soluble CD27 (sCD27), a molecule generated by proteolysis of membrane CD27 and directly involved in T cell activation [20]–[24]. Herein, we show that chronic Chagas cardiomyopathy but not asymptomatic *T. cruzi* infection, is associated with elevated levels of serum sCD27. Taken as a whole, our results demonstrate that chronic *T. cruzi* infection alters the distribution of various PB B cell subsets and increase serum sCD27, probably related to the T CD4+ cell immune deregulation process provoked by the parasite in humans.

## Materials and Methods

### Study population

A total of 72 participants were enrolled at the Instituto Nacional de Parasitología “Dr. M. Fatala Chaben” (INP), Buenos Aires, and Hospital Independencia, Province Santiago del Estero, Argentina. The clinical status of enrolled individuals was determined by physical examination and clinical tests, including serologic testing for *T. cruzi* infection, electrocardiography, chest radiography and echocardiography. Seropositive individuals with no clinical signs of heart failure (mean age ± SD = 42.1±12.5 years, n = 48) were included in the study and grouped according the Kuschnir clinical grading system [25]. A group of healthy uninfected controls (mean age ± SD = 36.7±12.4 years; n = 24) were recruited from the same geographical area as infected individuals. Age of controls was matched as much as made possible to that of the infected individuals for each assay. *T. cruzi*-infected subjects and controls with dilated cardiomyopathy [17], cancer, diabetes, autoimmune and lymphoproliferative disorders, HIV infection, syphilis, or serious allergies were excluded from this study. The study protocol was approved by the Institutional Review Board of the Instituto Nacional de Parasitología Dr Mario Fatala Chaben, Administración Nacional de Laboratorios e Institutos de Salud Dr. Carlos G. Malbran, and signed informed consent was obtained from all individuals before enrollment.

### Collection of PBMC and Immunophenotyping

PBMC were isolated by density gradient centrifugation on Ficoll-Paque Plus (GE Healthcare, Uppsala, Sweden) as described previously [5]. Frequencies of lymphocyte subsets were estimated in PBMC by means of flow cytometry using mAb against CD19-PE.Cy7, CD4-APC, CD27-FITC, CD10-APC, IgD-PE, IgM-PE.Cy5, IgG-APC and CD138-APC (BD Pharmingen, San Jose, CA, USA). Non-specific fluorescence was established with APC-mouse IgG1κ, PE-Cy7-mouse IgG1κ, FITC-mouse IgG1κ, PE-mouse IgG2aκ, PE-mouse IgG1κ isotype controls. At least 200,000 events per sample were collected in a FACSAria II flow cytometer (BD Immunocytometry Systems) and analyzed using FlowJo software (Tree Star, Inc., Ashland, OR, USA). Lymphocytes were identified by forward and side-angle light scatter characteristics. At least 80,000 lymphocyte-gated cells were analyzed for each sample. Absolute numbers were estimated based on the frequencies of the cells in flow cytometry and lymphocytes in the differential leukocyte count (Sysmex K-800 Automated Hematology Analyzer, Sysmex America, Inc., Mundelein, IL, USA), and expressed as the number of cells per microliter of blood.

### Determination of serum sCD27 levels

Serum levels of sCD27 were measured in duplicate samples by sandwich ELISA (Human sCD27 Instant ELISA kit, eBioscience, Vienna, Austria) following the instructions of manufacturers.

### Culture of PBMC for antibody secretion

A *T. cruzi* protein lysate was obtained from Brazil strain epimastigotes by 4 freeze/thaw cycles at −70°C, which was followed by sonication and centrifugation at 12,000 g. The supernatant was collected and filter-sterilized, and the protein concentration determined by Bradford technique. PBMC from 9 *T. cruzi*-infected individuals and 7 controls were suspended in RPMI-1640 supplemented with 10% FCS and seeded in 24-well tissue culture plates (2×10^6^ cells per well). Cells were stimulated with the *T. cruzi* lysate (25 µg/ml) or a cytokine cocktail containing recombinant human IL-2 (16 ng/ml), IL-6 (10 ng/ml) and IL-10 (17 ng/ml; BD Pharmingen) [26] for 5 days at 37°C with 5% CO2. PBMC incubated with 10% FCS-supplemented RPMI-1640 was used as non-stimulated controls. Supernatants were collected and kept at –20°C until testing.

### ELISA for *T. cruzi*-specific and total IgG determination

Ninety six-well flat-bottom plates were coated with *T. cruzi* Brazil strain lysate (5 µg/ml) or rabbit anti-human IgG (10 µg/ml; Dako, Denmark) overnight at 4°C. Plates were washed with PBS-Tween-20 and blocked with 5% dry, non-fat milk-PBS for 1 h at room temperature, followed by incubation with undiluted culture supernatants in duplicate wells for 45 minutes at 37°C. Plates were then incubated with 100 µl/well of HPR-labeled rabbit anti-human IgG (1∶1000; Dako) for 1 hour at 37°C. Color development was obtained by the addition of O-phenylenediamine (OPD, Sigma-Aldrich, Saint Louis, MO, USA) and read at 490 nm in an automatic ELISA reader (Bio-Tek Instruments, Inc., Winooski, VT, USA). Titers were calculated by subtracting the values obtained for the wells incubated with supernatants of unstimulated cultures from the values of wells incubated with supernatants of *T. cruzi* lysate- or cytokine cocktail- stimulated cultures. Selection of the cut-off point for *T. cruzi*-specific responses was based on the mean +3 SD of IgG titers obtained for lysate or cytokine cocktail stimulated supernatants in the control group, and responses were considered to be positive if the titer was higher than the cut-off value.

### Statistical analysis

Statistical analyses were performed using GraphPad Prism (GraphPad Software, Inc., La Jolla, CA, USA). The Kolmogorov-Smirnov normality distribution test was applied to the data previous analysis by ANOVA followed by Bonferroni or Kruskal-Wallis followed by Dunns for multiple comparisons, Unpaired Student T-test or Mann-Whitney test, and Spearman or Pearson tests for correlations. A simple linear regression test was used for trend analysis. Differences were considered statistically significant when p<0.05.

## Results

### Major B cell subsets are differentially affected by chronic *T. cruzi* infection

**Figure 1:**
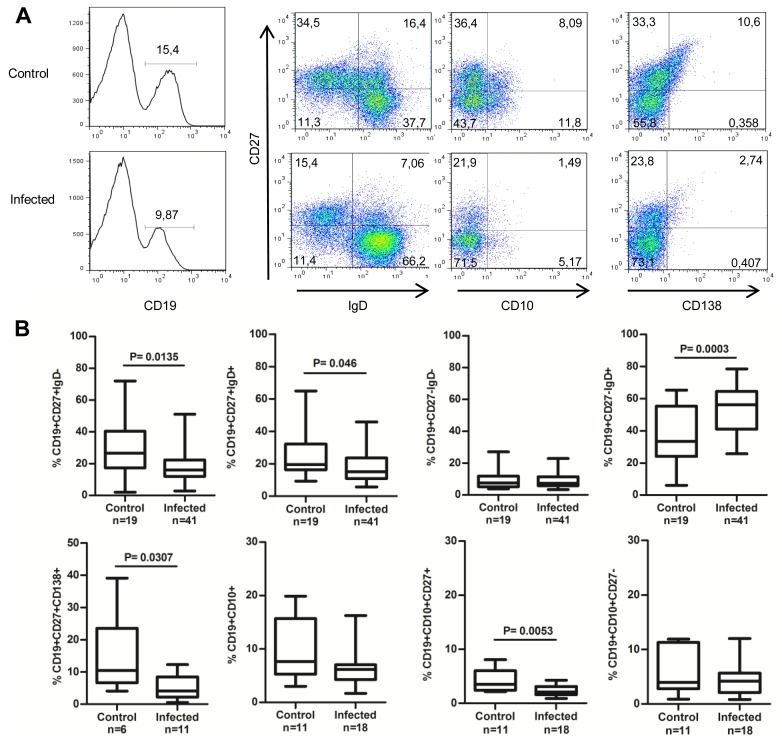
Distribution of the major circulating B cell subsets in individuals chronically infected with *Trypanosoma cruzi*. (A) Representative samples from a healthy control donor and a *T. cruzi*-infected individual. PBMC were stained with Abs to CD19, CD27, IgD, CD10 and CD138 and analyzed by flow cytometry. Plots show a typical staining for CD19+ cells (histogram) and major B cell subsets among gated CD19+ cells. Numbers represent the percentage of cells within the gate. (**B**) Box and whiskers (min to max) show the percentage of cells expressing the indicated markers. Mann-Whitney nonparametric test or U-test was used for statistical significance (p values).

In order to evaluate whether *T. cruzi* infection affects the phenotype of the PB B cell pool in humans, we first determined the overall proportion of B cells by staining PBMC from 48 *T. cruzi*-infected individuals and 24 uninfected controls with anti-CD19 (Fig. 1A). The proportion of CD19+ cells in infected individuals was significantly lower compared to controls (mean percentage ± SD = 12.53±6.37 and 16.86±7.48, respectively; P = 0.0177). However, absolute numbers of CD19+ cells, estimated for a subset of infected individuals, were found comparable to that observed in controls (Table 1). We then evaluated the major circulating B cell subsets based on the differential expression of CD27 and IgD on CD19+ cells (Fig. 1A). *T. cruzi*-infected individuals had significantly lower frequencies of CD27+IgD- and CD27+IgD+ B cells, and higher frequencies of CD27-IgD+ B cells compared to controls (p<0.05); the levels of CD27-IgD- B cells in infected individuals were comparable to that in controls (Fig. 1B).

**Table 1:** Absolute numbers of cells of various phenotypes in the peripheral blood of individuals chronically infected with *Trypanosoma cruzi*.

Cell phenotype	Control	Infected
	(n = 6)	(n = 10)
Lymphocytes	2288 (2220–2457)	2346 (2044–2546)
CD19+	347 (219–615)	301 (160–367)
CD19+CD27+	115 (96–164)	64 (45–86)*
CD19+CD27+CD138+	7 (5–24)	4 (3–5)*
CD19+CD10+	48 (20–120)	16 (12–28)
CD19+CD10+CD27+	14 (8–50)	5 (3–6)*
CD19+CD10+CD27−	34 (12–70)	13 (8–22)
CD19+CD10−CD27−	205 (96–268)	219 (99–255)
CD4+	876 (633–950)	756 (553–1051)
CD4+CD27+	764 (554–822)	585 (314–735)

Data represent median cell numbers/µL blood (interquartile range) for each phenotype.

*p<.05 vs. Controls; Mann-Whitney or U-test.

### *Trypanosoma cruzi* provokes a selective reduction of CD27+ memory B cells

**Figure 2:**
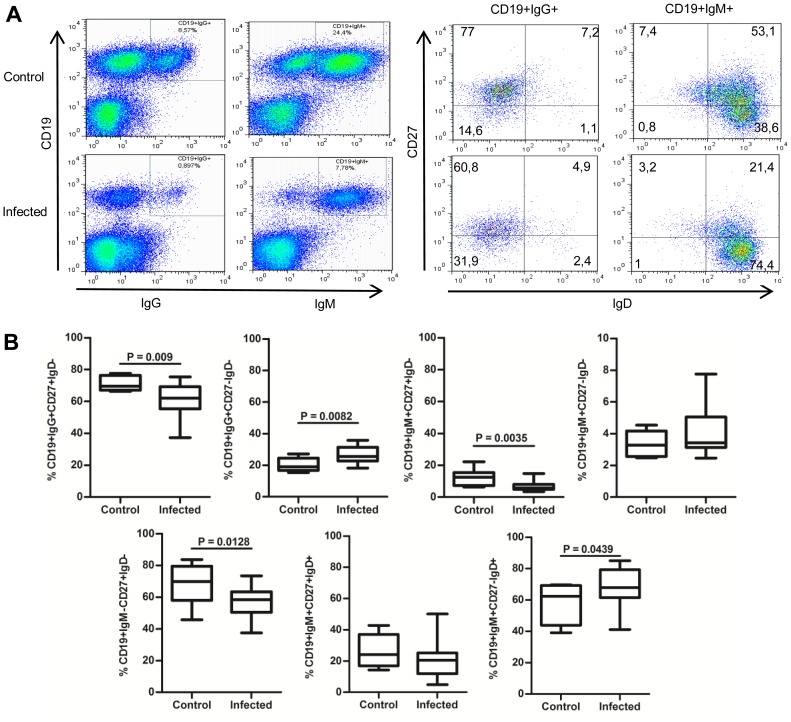
Chronic infection with *Trypanosoma cruzi* modulates circulating memory B cell subsets. (**A**) Representative samples from a control donor and a *T. cruzi*-infected individual. PBMC were stained with Abs to CD19, CD27, IgD, IgM and IgG, and analyzed by flow cytometry. Numbers correspond to the percentage of cells within the gate. (**B**) Box and whiskers (min to max) show the percentage of cells expressing the indicated markers for control (n = 8) and infected individuals (n = 18). Groups were compared by the Mann-Whitney nonparametric test or U-test and statistical significance (p values) is indicated.

To determine the relative contribution of memory B cells of different isotypes on peripheral B cells, we delineated IgG+ (IgG+CD27+IgD−), IgM-only (IgM+CD27+IgD−) and isotype-switched (IgM-CD27+IgD−) memory B cells (Fig. 2A) [27]–[30]. There was a significant reduction in the proportion of IgG+CD27+IgD-, IgM+CD27+IgD- and IgM-CD27+IgD- memory B cells in *T. cruzi*-infected individuals compared to controls (p<0.05; Fig. 2B). Conversely, the proportions of IgG+CD27-IgD- B cells were significantly higher in infected individuals respect to controls (p<0.05). The levels of IgM+CD27-IgD- B cells in infected individuals were comparable to that of controls.

### Reduced frequency of plasma cells in *Trypanosoma cruzi* infected individuals

Next, we sought to extend our analysis to terminally differentiated plasma cells, which were identified by the expression of CD138 on CD19+CD27+ cells (Fig. 1A) [31]. *T. cruzi*-infected individuals had a significant reduction of CD19+CD27+CD138+ cells, in terms of proportions and absolute numbers compared to controls (p<0.05; Fig. 1B and Table 1). No correlation was found between the frequencies of plasma cells and CD19+, CD19+CD27+IgD- or CD19+CD27-IgD- B cells, or serum anti-*T.cruzi* IgG levels as measured by conventional ELISA (data not shown).

### “Innate-like” IgM+CD27+IgD+ memory B cells

The expression of IgM on CD19+CD27+IgD+ cells allowed us to delineate a population of B cells with mutated polyclonal Ig repertoire that comprises at least three functionally distinct subpopulations, marginal zone-like B cells (MZ-like), regulatory B cells and B1 cells [32]–[37]. The origin of these cells is still a matter of discussion. The analysis of CD19+IgM+CD27+IgD+ cells in our study showed no differences in the proportions of these cells between *T. cruzi*-infected individuals and controls (Fig. 2B), suggesting that this population is not particularly affected by the infection. Nevertheless, a positive correlation between the proportions of CD19+IgM+CD27+IgD+ and CD19+CD27+IgD+ B cells was found in infected individuals (r = 0.727, P = 0.007), but not in controls (r = 0.533, P = 0.139).

### Transitional/Naïve IgM+CD27-IgD+ B cells

To assess whether *T. cruzi* affects earlier developmental stages of the human B cell maturation process, we next analyzed CD19+IgM+CD27-IgD+ cells that comprise transitional and mature naïve B cells. The proportion of CD19+IgM+CD27-IgD+ cells in infected individuals was significantly increased compared to controls (p<0.05; Fig. 2B). To define immature/transitional B cells, we analyzed the expression of CD10 (a classical marker of bone marrow B cells) on total CD19+ cells (Fig. 1A) [38], [39] and found no significant difference in the frequency and absolute number of CD19+CD10+ B cells between infected individuals and controls (Fig. 1B and Table 1). Besides being a marker for activated B cells, CD27 is also expressed by a small population of CD19+CD10+ B cell precursors [34], [40]. In our study, infected individuals had significantly reduced proportions and absolute numbers of CD10+ CD27+ (p<0.05), but not CD10+CD27− B cells compared to controls (Fig. 1B and Table 1).

On the whole, our data demonstrate that chronic exposure to *T. cruzi* alters the distribution of selective circulating B cells, at different levels of the maturation process.

### Correlation between CD19+CD27+ and CD4+CD27+ cells

**Figure 3:**
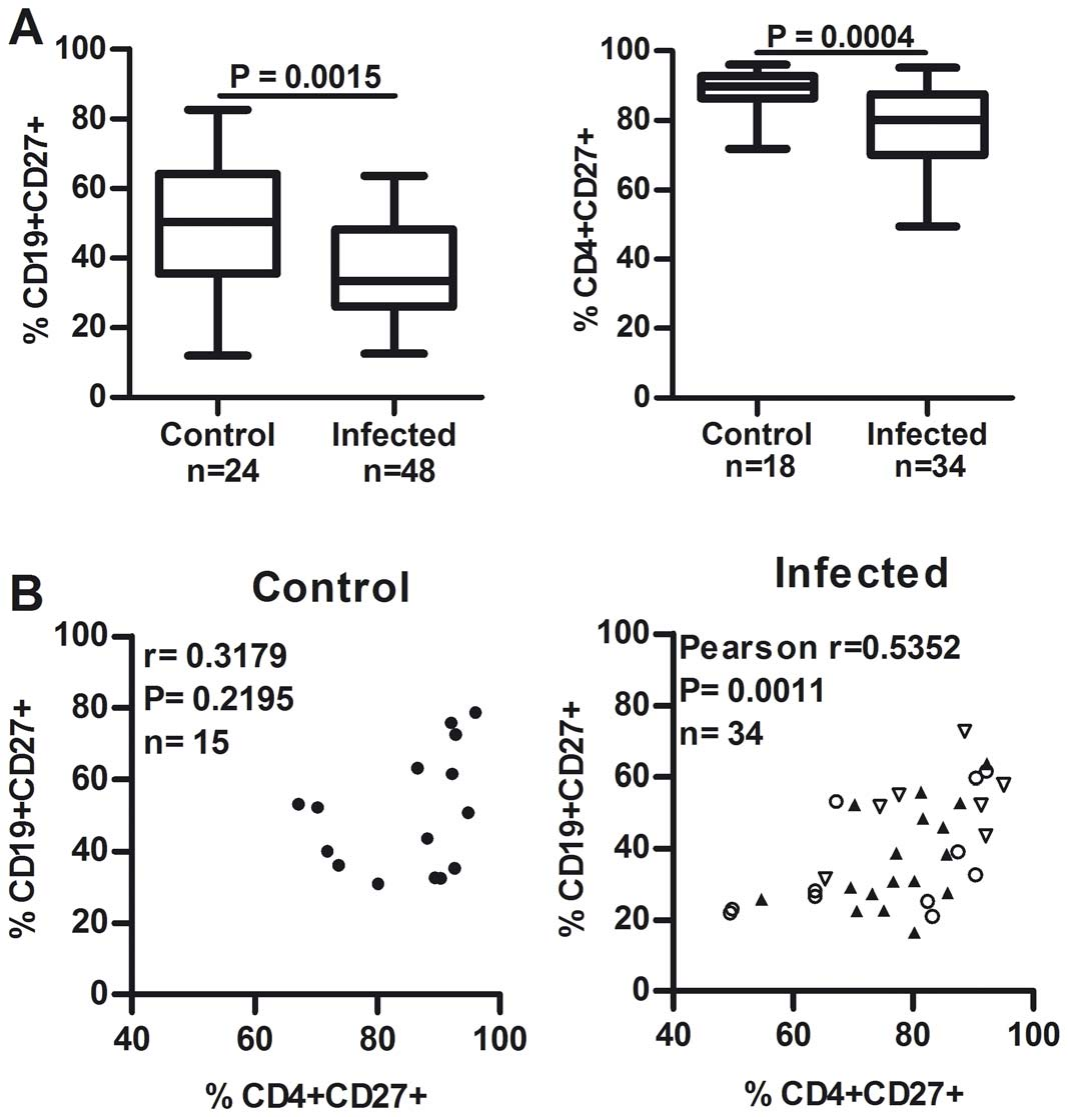
Reduced proportions of circulating CD19+CD27+ and CD4+CD27+ cells in *Trypanosoma cruzi-* infected individuals. (**A**) Box and whiskers (min to max) show the percentage of cells expressing the indicated markers for controls and infected individuals. Groups were compared by the Mann-Whitney nonparametric test or U-test (p values). (**B**) Expression of CD27 on CD19+ cells is graphed as function of CD4+CD27+ T cell frequency in controls (filled circles) and *T. cruzi*-infected individuals with normal ECG and chest X-rays (G0, empty circles), infected individuals with electrocardiographic abnormalities associated to chronic Chagas heart disease and normal chest X-rays (G1, filled triangles) and patients with cardiomegaly (G2, non-dilated chagasic cardiomyopathy, empty triangles). Data correlation was determined with Spearman test.

CD4+ helper T cells are known to play a crucial role in the development of effective B cell responses [27], [41]. We here examined the expression of CD27 on circulating CD4+ T cells, known to be up-regulated upon activation via the TCR/CD3 complex [21]. There was a significant reduction in the proportion of CD4+CD27+ T cells in infected individuals compared to controls (p<0.05, Fig. 3A). The proportion of CD4+CD27+ cells positively correlated with a decreased frequency of CD19+CD27+ cells in infected individuals (p<0.05) but not in controls (Fig. 3B), suggesting that a weakened CD4+ T cell activation might account, at least in part, for the abnormal distribution of the PB B cell pool.

To dissect the relationship between altered CD27+ B cell frequencies and disease progression, immunologic data was further analyzed according to the clinical classification of Kuschnir [25], namely individuals with long-term non-progressive infection (G0), individuals with electrocardiographic abnormalities associated to chronic Chagas heart disease (G1) and patients with non-dilated chagasic cardiomyopathy (G2). Individuals in G0 had significantly lower proportions of CD19+CD27+ cells compared to individuals in G2 (mean percentage ± SD = 32.43±13.69 and 48.60±9.65, respectively; P = 0.032). Linear regression analysis evidenced a positive trend for these cells as Chagas heart involvement increases (P = 0.0168), suggesting that the contraction of CD19+CD27+ cells occurs prior the onset of heart symptoms, and reverts as cardiac disease becomes more severe. There was no statistically significant difference in the proportions of any of the other PB CD19+ cell subpopulations analyzed or CD4+CD27+ T cells among the clinical groups.

### sCD27 as a potential immunological marker for chronic Chagas cardiomyopathy

**Figure 4:**
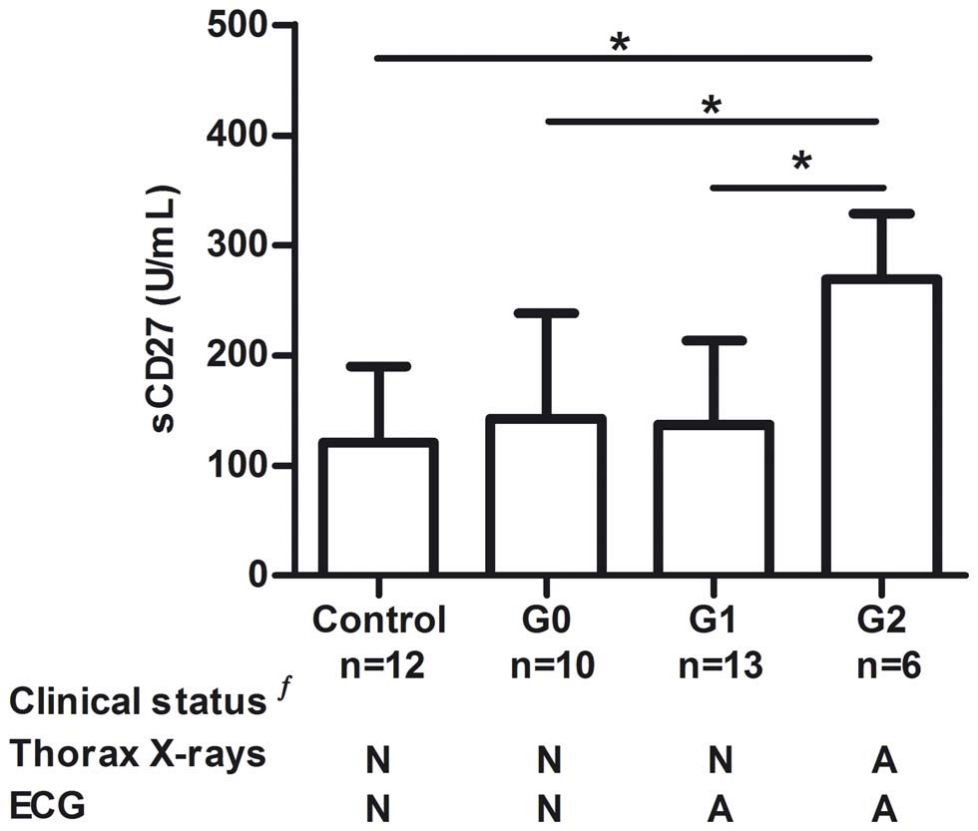
Comparison of serum sCD27 levels in individuals chronically infected with *Trypanosoma cruzi*. The levels of sCD27 were measured by ELISA in individuals with different degrees of cardiac dysfunction and controls. Bars represent the mean and SD for each group. Statistical significance was determined with One-way ANOVA followed by Bonferroni’s post-test. *p<0.05. **^ƒ^** N, normal and A, abnormal.

sCD27 has been related to membrane CD27 expression on activated lymphocytes, and used to monitor systemic immune activation in a variety of immunological disorders such as chronic viral infections, autoimmune diseases and B cell malignancies [22], [23], [42], [43]. Here we measured the levels of serum sCD27 in *T. cruzi*-infected individuals with different degrees of cardiac dysfunction. Individuals with Chagas cardiomyopathy had increased levels of sCD27 as compared to the other groups of infected subjects and controls (P = 0.0034; Fig. 4). No correlation between the levels of sCD27 and CD19+CD27+ or CD4+CD27+ cells was found. The observation of elevated levels of sCD27 in association with Chagas cardiomyopathy is of great interest for monitoring disease progression in endemic areas of Chagas disease, as the ELISA technique used to determine the levels of sCD27 is much simpler to perform than flow cytometry.

### *In vitro* antibody production

**Figure 5:**
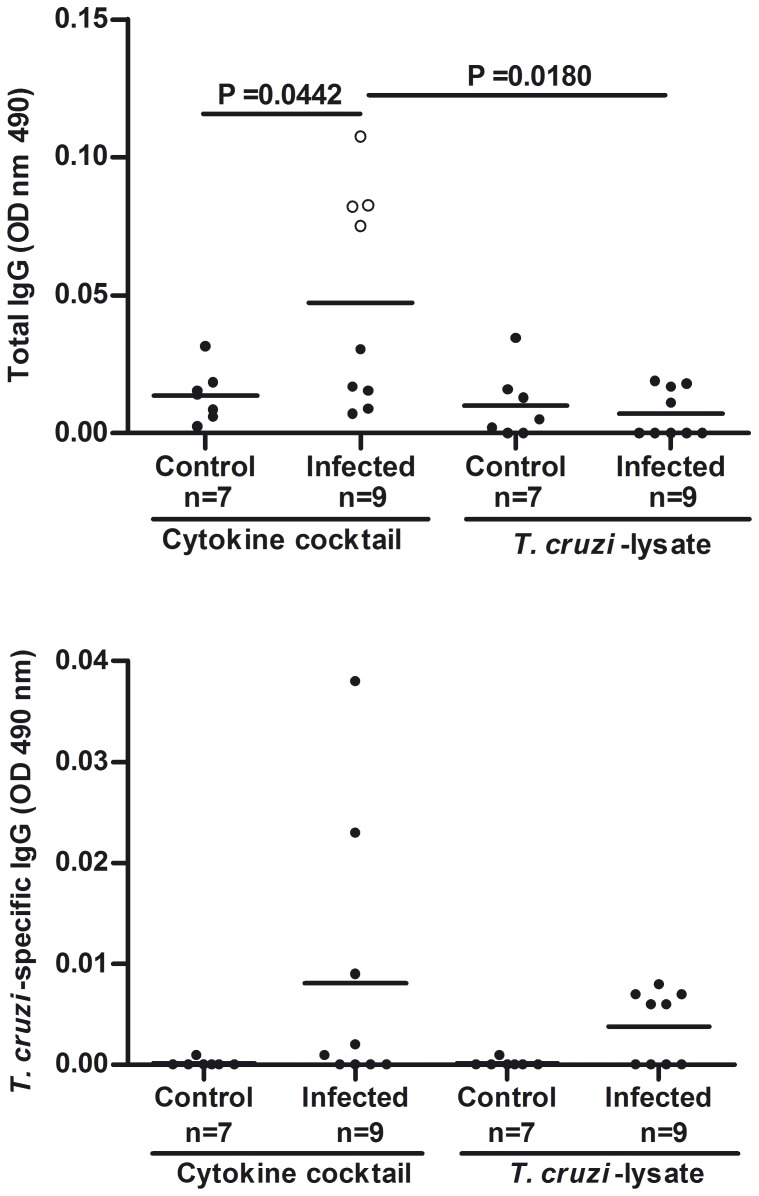
*In vitro* secretion of IgG by PBMC of individuals chronically infected with *T. cruzi*. The levels of *T. cruzi*-specific and total IgG were measured by ELISA in supernatants of PBMC from *T. cruzi*-infected individuals and healthy controls, stimulated with a cytokine cocktail or a parasite lysate for 5 days. Each mark represents an individual; open marks indicate positive parasite-specific IgG responses and filled marks non-responders. Groups were compared by the Mann-Whitney nonparametric test or U-test, and statistical significance (p values) is indicated.

In order to evaluate the ability of circulating B cells to secrete IgG in response to non-specific and *T. cruzi*-specific stimulation in vitro, we incubated PBMC with a cytokine cocktail or an epimastigote lysate, and measured total and parasite-specific IgG levels in the supernatants by ELISA. An in-house Brazil strain antigen preparation, shown to be useful for studying parasite specific antibody responses in humans [44], was used as an experimental tool to detect anti-*T. cruzi* IgG in supernatants. Stimulation with cytokines led to significantly higher titers of total IgG in cultures of PBMC from infected individuals as compared to controls and to stimulation with the lysate (p<0.05, Fig. 5). There were no differences in the levels of total IgG induced by stimulation with the parasite lysate between infected individuals and controls (Fig. 5). The levels of *T. cruzi*-specific IgG were low (OD<0.05), regardless the infection status of the individuals and stimulation condition. These data support the notion that a large proportion of the IgG secreted in vitro by PBMC from Chagas disease patients is not specific for the parasite reported by other authors [12], [13].

## Discussion

This is the first report on the effect of *T. cruzi* infection on the major circulating B cell subsets in humans. We show that, although absolute B cell numbers were not significantly altered in chronically infected individuals, a selective reduction of circulating CD27+ B cells but not CD27− B cells are seen. Further characterization of the circulating B cell pool revealed that the decreased frequencies of CD27+ B cells resulted from the combined reduction of IgG+, IgM-only and isotype switched memory B cells, CD10+CD27+ B cell precursors and terminally differentiated CD19+CD138+CD27+ plasma cells. The constraint of various kinds of CD27+ B cell subpopulations affecting different levels of the B cell maturation process suggests a common mechanism behind this phenomenon. The attenuated phenotype of the peripheral B cell pool contrasts with the sustained levels of parasite specific antibodies found in the serum of these individuals. However, when PBMC from infected individuals were cultured for 5 days to allow the existing memory B cell populations to proliferate and differentiate to plasma cells, we were unable to detect a significant parasite-specific IgG antibody response in vitro. On the whole, our data demonstrates that *T. cruzi* provokes a narrowing of the peripheral CD27+ B cell pool, and memory B cells that persist in the circulation are not competent to generate a substantial *T. cruzi*-specific IgG response.

We previously reported that chronic *T. cruzi* infection led to defective parasite-specific IFN-γ T cell responses along with a contraction of peripheral early-differentiated memory (CD27+CD28+CD45RA−) and naïve-like (CD27+CD28+CD45RA+) T cells in humans [5]. It is conceivable that inadequate CD4+ T cell activity during chronic *T. cruzi* infection may contribute to a defective parasite-specific humoral immunity. Besides their essential role in antibody production against foreign antigens, B cells also perform other functions such as cytokine secretion, presentation of antigens and providing co-stimulatory signals to T cells [18], [27], [33], [41], [45]. The interaction of CD27 with its ligand CD70 (expressed on the surface of activated lymphocytes) provides a co-stimulatory signal for T and B cell expansion and acquisition of effector function [46], [47]. As the contraction of CD27+ B cells precedes the onset of clinical symptoms and the loss of *T. cruzi*-specific IFN-γ-secreting T cells is associated with the more severe forms of Chagas heart disease, it is also possible that altered B cells are the reason for some of the deregulated T cell responses [19]. Even though the reduced CD4+CD27+ and CD19+CD27+ cells observed in the present study suggest a causal relationship, the correlation between them might simply reflect the fact that both populations are driven by a common factor.

*T. cruzi* species comprises a highly heterogeneous population of parasites. Variations among *T. cruzi* parasites were shown to influence the virulence and pathogenic characteristics of the isolates [48]. Parasite strains were classified into discrete typing units (DTUs; I-VI), associated with different geographical distribution and transmission cycles [49]. Genetic diversity of the parasite was demonstrated to correlate with the pattern of humoral immune responses [50] and the outcome of human Chagas disease [49]. Antibody responses elicited by *T. cruzi* infection are capable of controlling parasitemia levels, but ineffective to completely eliminate the parasite from the organism. Some of the antibodies directed against the parasite cross react with host proteins, suggesting their potential role in the pathogenesis of chronic Chagas heart disease [51], [52]. Moreover, antibodies specific for *T. cruzi* calreticulin (TcCRT), a highly conserved molecule expressed also in humans, rabbits and mice, were demonstrated to enhance the infectivity of the parasite and thereby increase its virulence [53]. Classically, 2 types of *T. cruzi* specific antibodies have been described, those capable of neutralizing live trypomastigotes (lytic antibodies), predominantly IgG1 and IgG3 subclasses, potent activators for the complement system and inducers of antibody-dependent cell-mediated-cytotoxicity, and antibodies reacting with fixed epimastigotes antigen preparations, that persist long after the elimination of parasites [54], [55]. Interestingly, a dominant use of IGHG1 and IGHG3 genes and mRNA expression for IgG1 and IgG3 were described for CD19+ IgG+CD27-IgD- memory B cells of humans [29], [30], [56]. We here show an expansion of CD19+IgG+CD27-IgD- memory B cells in *T. cruzi* infected individuals, suggesting a potential role of these cells in the production of specific lytic antibodies.

Other authors have reported the depletion of parasite-specific IgG-producing B cells through a Fas/Fas-L mechanism during the acute infection of mice, which were suggested to hamper B cell responses and favor parasite replication and chronicity [57]. Double expression of Fas and Fas-L associated with apoptosis were also reported for PBMC of chronically infected humans, in association with high production of TNF-α and low proliferative responses [58]. Thus, it is conceivable that apoptosis of activated specific lymphocytes [59] in chronically infected individuals contributes to the narrowing of the circulating CD27+ memory B cell pool. On the other hand, cleavage of membrane-bound CD27 from activated memory B cells by the action of MMPs [60], [61] seems unlikely to play a major role in the shaping of the PB B cell compartment during the early stages of chronic infection, as sCD27 serum levels remain essentially unchanged prior the development of Chagas cardiomyopathy. Finally, we cannot rule out that some of the reduction of circulating CD27+ cells may result from down-regulation of CD27.

In summary, our study identified altered homeostasis in the PB B cell compartment of individuals with chronic *T. cruzi* infection. An early and effective B cell response is essential for clearing pathogenic microorganisms. It is plausible that the reduction of memory B cells and plasma cells account for the defective antibody response that impairs the elimination of parasites that induce Chagas disease, resulting in parasite persistence and chronic inflammation. A limitation of our study is that concurrent *T. cruzi* DTU was not determined in the study population and thus, we cannot establish the relationship between B cell defects and the lineage of the infecting parasite. A prospective study is now underway to define *T. cruzi* variants circulating in human Chagas disease patients in our region, and their association with B cell responses.
